# Best Practices for Measuring the Social, Behavioral, and Economic Impact of COVID 19 Using Secondary Data

**DOI:** 10.1093/geroni/igaa057.3521

**Published:** 2020-12-16

**Authors:** James McNally, Kathryn Lavender

**Affiliations:** University of Michigan, Ann Arbor, Michigan, United States

## Abstract

This presentation reviews best practices for using data resources from the NACDA Program on Aging, its projects, and its collaborating partners for measuring the impact of epidemics. The report summarizes resources to identify measures of well-being, social connectedness, and other constructs to measure the social and behavioral effects of the COVID-19 epidemic on population health outcomes. The report suggests data resources to identify pre-crisis measures of social distancing, social networks, consumer confidence, unemployment, and the use of social media. The COVID-19 pandemic presents research challenges for how we measure governmental, community, and population responses to a crisis. COVID-19 represents a unique case because it is a new virus with a novel impact profile and a long latency period. We offer examples of variables and concepts found across multiple studies and multiple years that can provide baseline information on pre-COVID-19 social behaviors using studies managed and distributed by NACDA and ICPSR. This report also provides guidance on how to search for specific topics using the ICPSR generated search tools. Finally, we offer information on how to obtain these data for research purposes. Our hope is this information will be valuable in identifying key baseline measures of social behavior, health, and employment before the COVID-19 epidemic, and this information can allow us to evaluate challenges and recovery once the crisis has passed. Both NACDA and ICPSR remain available 24 hours a day, 365 days a year, to assist researchers in locating and obtaining data related to their research.

